# Invasive electrophysiological testing to predict and guide permanent pacemaker implantation after transcatheter aortic valve implantation: A meta-analysis

**DOI:** 10.1016/j.hroo.2022.10.007

**Published:** 2022-10-22

**Authors:** Konstantinos C. Siontis, Abdalla Kara Balla, Yong-Mei Cha, Thomas Pilgrim, Romy Sweda, Laurent Roten, Tobias Reichlin, Paul A. Friedman, Stephan Windecker, George C.M. Siontis

**Affiliations:** ∗ Department of Cardiovascular Medicine, Mayo Clinic, Rochester, Minnesota; † Department of Cardiology, Bern University Hospital, University of Bern, Bern, Switzerland

**Keywords:** Aortic stenosis, Electrophysiological study, Permanent pacemaker, Risk stratification, Transcatheter aortic valve implantation

## Abstract

**Background:**

Atrioventricular conduction abnormalities after transcatheter aortic valve implantation (TAVI) are common. The value of electrophysiological study (EPS) for risk stratification of high-grade atrioventricular block (HG-AVB) and guidance of permanent pacemaker (PPM) implantation is poorly defined.

**Objective:**

The purpose of this study was to identify EPS parameters associated with HG-AVB and determine the value of EPS-guided PPM implantation after TAVI.

**Methods:**

We performed a systematic review and meta-analysis of studies investigating the value of EPS parameters for risk stratification of TAVI-related HG-AVB and for guidance of PPM implantation among patients with equivocal PPM indications after TAVI.

**Results:**

Eighteen studies (1230 patients) were eligible. In 7 studies, EPS was performed only after TAVI, whereas in 11 studies EPS was performed both before and after TAVI. Overall PPM implantation rate for HG-AVB was 16%. AV conduction intervals prolonged after TAVI, with the AH and HV intervals showing the largest magnitude of changes. Pre-TAVI HV >70 ms and the absolute value of the post-TAVI HV interval were associated with subsequent HG-AVB and PPM implantation with odds ratios of 2.53 (95% confidence interval [CI] 1.11–5.81; *P* = .04) and 1.10 (95% CI 1.03–1.17; *P* = .02; per 1-ms increase), respectively. In 10 studies, PPM was also implanted due to abnormal EPS findings in patients with equivocal PPM indications post-TAVI (typically new left bundle branch block or transient HG-AVB). Among them, the rate of long-term PPM dependency was 57%.

**Conclusion:**

Selective EPS testing may assist in the risk stratification of post-TAVI HG-AVB and in the guidance of PPM implantation, especially in patients with equivocal PPM indications post-TAVI.

**Key Findings**

▪ The rate of high-grade atrioventricular block (HG-AVB) after transcatheter aortic valve implantation (TAVI) is consistent with previous literature.
▪ The HV interval before and after TAVI showed the most consistent association with risk of HG-AVB.
▪ In studies in which a permanent pacemaker (PPM) was implanted due to abnormal electrophysiological study (EPS) findings in patients with equivocal PPM indications after TAVI, the rate of PPM dependency at long-term follow-up was 50%.
▪ Selective use of EPS testing, especially among patients with equivocal PPM indications, may be helpful in risk stratification for HG-AVB after TAVI.

## Introduction

Transcatheter aortic valve implantation (TAVI) is now a mainstream approach for the treatment of severe aortic stenosis in elderly patients.^1^^,^^2^ The number of patients undergoing TAVI is predicted to continue to grow.^3^^,^^4^ Although high valve frame implantation techniques directly resulted in a significant decrease in the rate of permanent pacemaker (PPM) implantation,^5^ atrioventricular (AV) conduction disturbances after TAVI remain an important limitation.^6^ Despite several known risk factors for post-TAVI PPM requirement,^7^^,^^8^ some patients with normal pre-TAVI conduction system still are at risk for high-grade atrioventricular block (HG-AVB). Furthermore, post-TAVI HG-AVB may occur with latency beyond the immediate postprocedural period,^9^ and some new-onset conduction abnormalities, such as left bundle branch block (LBBB), represent management challenges due to uncertainty about the risk of progression to complete AVB.^10^^,^^11^ Therefore, identifying patients at risk for persistent HG-AVB post-TAVI remains challenging.^12^

To address these challenges, various invasively measured electrophysiological (EP) parameters of the conduction system have been investigated as potential predictors of post-TAVI HG-AVB and PPM requirement. However, results have been largely inconclusive, and data supporting the routine use of electrophysiological studies (EPS) for risk stratification of patients undergoing TAVI are sparse.

The aims of this systematic review and meta-analysis were (1) to synthesize the available evidence on the value of peri-TAVI EPS parameters in the risk stratification of post-TAVI HG-AVB; and (2) to determine their value in guiding PPM implantation among patients with equivocal PPM indications post-TAVI.

## Methods

The study protocol follows the Preferred Reporting Items for Systematic Reviews and Meta-Analyses statement^13^ and was registered at the PROSPERO international register of systematic reviews (CRD42019121204). Institutional REVIEW BOARD approval was not required due to the nature of the study, which utilized published data only.

### Search sources and strategy

We searched PubMed, Embase, and the Cochrane Central Register of Controlled Trials using a prespecified search algorithm for each database (Supplemental Material Section 1). After the initial search, we scrutinized the reference lists of potentially eligible articles for relevant entries. Articles published up to March 15, 2022, were considered for inclusion in this systematic review and meta-analysis without language restriction.

### Eligibility criteria and study selection

We included 2 types of original prospective or retrospective studies: those investigating the association of EPS-derived parameters with HG-AVB and PPM requirement after TAVI; and those investigating an EPS-guided approach to PPM implantation among patients with equivocal PPM indications (without HG-AVB) after TAVI. Further inclusion criteria included TAVI performed for severe stenosis of native aortic valve; EPS performed before and/or after TAVI; and available quantitative data for any EPS parameters before and/or after TAVI, rates of PPM implantation after TAVI, or outcomes of EPS-guided PPM implantation among patients with equivocal post-TAVI PPM indications. We did not apply any restrictions on the study-level number of enrolled patients, type of valve prosthesis, or minimum follow-up duration. Two independent investigators screened search results on title and abstract level and assessed the studies for eligibility in full text. Disagreements between reviewers were resolved by arbitration by a third reviewer.

### Data extraction

Two investigators independently reviewed the full text and any supplementary material of eligible studies and extracted study-level data into an electronic data abstraction form. Disagreements were resolved by consensus. We summarized the timing of EPS relative to TAVI and the indications and timing (days after TAVI) of PPM implantation. We documented changes of EPS-derived parameters of AV conduction after TAVI. We also extracted any available crude or adjusted risk association estimates (with corresponding 95% confidence interval [CI]) for each EPS-derived parameter as a predictor of PPM requirement. Furthermore, for studies in which patients with equivocal post–transcatheter aortic valve replacement (TAVR) AV conduction abnormalities underwent PPM implantation due to abnormal EPS findings (rather than HG-AVB), we documented the rates of pacemaker dependency, as defined in each study, at the time of post-TAVI follow-up. We also documented rates of sudden cardiac death and subsequent incident PPM implantations among patients with a negative EPS who did not receive a PPM early after TAVI.

### Data synthesis

We visualized the changes in the mean values of the EPS parameters before and after TAVI in paired box plots. We quantified the magnitude of the changes in the mean values of EPS parameters before and after TAVI with the standardized effect size (Cohen's d). The changes were considered small, medium, and large for absolute values of Cohen’s d of 0.2–0.5, 0.5–0.8, and >0.8, respectively. We quantified the variance and calculated the SD of the standardized effect sizes. For data reported as median [interquartile range] or 95% CI, we calculated mean ± SD as previously described.^14^

Meta-analysis was performed when at least 2 studies reported the same EPS parameter of interest; otherwise, data were reported only descriptively. We applied random-effects meta-analysis models to summarize crude or adjusted effect estimates of EPS parameters as predictors of PPM implantation due to HG-AVB. We gave preference to adjusted over unadjusted estimates. The summary association metric in the meta-analysis was the odds ratio (OR), and any required transformations were performed as previously described.^15^^,^^16^ We used random-effects meta-analyses with Hartung-Knapp-Sidik-Jonkman adjustments due to the relatively small number and heterogeneous studies.^17^^,^^18^ Heterogeneity was assessed by τ^2^, and the estimator was based on the restricted maximum-likelihood method.^19^ Values of τ^2^ approximating 0.04, 0.16, and 0.36 were considered to represent low, moderate, and high heterogeneity, respectively.^20^ R Version 4.0.2 was used for all analyses.

## Results

### Study characteristics

The literature search identified 657 potentially eligible studies, of which 24 were further evaluated in full text. A total of 18 studies^21^, ^22^, ^23^, ^24^, ^25^, ^26^, ^27^, ^28^, ^29^, ^30^, ^31^, ^32^, ^33^, ^34^, ^35^, ^36^, ^37^, ^38^ with 1230 patients (mean 68 patients per study) reporting on nonoverlapping patient populations were considered eligible (Supplemental Material Section 2). The characteristics of the included studies and patients are summarized in Tables 1 and 2. Thirteen studies were prospective, and 1 was multicenter. Most studies (n = 13) included patients without pre-existing PPM undergoing peri-TAVI EPS, whereas 5 studies were restricted only to patients undergoing EPS because of an equivocal indication for PPM after TAVI, such as transient HG-AVB and new LBBB. Seven studies included only self-expanding prostheses, and 11 studies included both self-expanding and balloon-expandable prostheses. In 11 studies, EP testing was performed both before and after TAVI, and in 7 studies it was only performed after TAVI. Pre-TAVI EPS typically was performed immediately before valve deployment. Timing of post-TAVI EP testing ranged from immediately post–valve deployment to 7 days later. Reported follow-up ranged from 2 days to 30 months after the index hospitalization.

**Table 1:** Baseline characteristics of the included studies

Study	Publication year	Enrollment period	Design	Centers	No. of patients	Age (y)	Male [n (%)]	Self-/balloon-expandable prosthesis [n (%)]	Follow-up
EPS performed in all patients for prognostication before and/or after TAVI									
Rubin et al^21^	2011	Dec 2009–Aug 2010	Prospective	Single	18	85 ± 3	4 (22)	18 (100)/0	12 mo
Akin et al^22^	2012	Jan 2007–Jan 2008	Retrospective	Single	45	82 ± 7	18 (40)	45 (100)/0	6 mo
Eksik et al^23^	2013	Oct 2010–Feb 2012	Prospective	Single	28	78 ± 5	11 (39)	28 (100)/0	2 d
Rivard et al^24^	2015	Jan 2009–Jul 2012	Prospective	Single	75	82 ± 7	48 (64)	64 (85)/11 (15)	24 mo
Shin et al^25^	2015	Oct 2011–Mar 2012	Prospective	Single	25	N/A	N/A	25 (100)/0	10 mo
Eksik et al^26^	2016	Jun 2012–Mar 2016	Prospective	Single	55	77 ± 7	23 (42)	25 (45)/30 (55)	5 mo
Kostopoulou et al^27^	2016	Jan 2010–Feb 2012	Prospective	Single	30	81 ± 5	18 (60)	30 (100)/0	17 mo
Lopez-Aguilera et al^28^	2016	Ap 2008–Dec 2013	Prospective	Single	131	78 ± 5	60 (46)	131 (100)/0	5 d
Badenco et al^30^	2017	Jan 2013–Dec 2014	Prospective	Single	84	83 ± 9	34 (41)	56 (67)/28 (33)	7 d
Makki et al^31^	2017	Nov 2011–Jan 2016	Retrospective	Single	7	N/A	N/A	5 (71)/2 (29)	3 mo
Krishnaswamy et al^34^	2020	Jan 2016–Aug 2018	Prospective	Multicenter	284	81 [75–85]	154 (54)	68 (24)/216 (76)	1 mo
Reiter et al^35^	2020	Jan 2017–Jan 2019	Prospective	Single	108	80 ± 5	42 (39)	108 (100)/0	1 mo
Ferreira et al^38^	2021	Jun 2018–Jul 2019	Prospective	Single	74	82 ± 6	36 (48)	35 (48)/39 (52)	3–6 mo
EPS performed in patients with equivocal pacing indication post-TAVR									
Tovia-Brodie et al^29^	2016	Mar 2009–May 2015	Retrospective	Single	26	82 [65–94]	10 (39)	19 (73)/7 (27)	12 mo
Rogers et al^32^	2018	Jan 2013–Dec 2015	Prospective	Single	95	80 ± 9	51 (54)	47 (49)/48 (51)	30 mo
Knecht et al^33^	2020	N/A	Prospective	Single	56	82 ± 6	23 (41)	40 (71)/16 (29)	12 mo
Bourenane et al^36^	2021	Jun 2017–Jul 2020	Retrospective	Single	78	84 [80–86]	48 (61)	15 (20)/63 (80)	5 mo
Nauchi et al^37^	2021	Jun 2019–Oct 2020	Retrospective	Single	11	87 ± 8	1 (9)	6 (54)/5 (46)	3 mo

Values are given as absolute number (n) with percentage (%), mean ± SD, or median [interquartile range] as reported in the primary studies.

EPS = electrophysiological study; N/A = not applicable/available; TAVI = transcatheter aortic valve implantation; TAVR = transcatheter aortic valve replacement.

**Table 2:** Study-level inclusion criteria, criteria for permanent pacemaker implantation, and timing of EPS

Study	Patient population	Criteria for PPM	Timing of EPS
EPS performed in all patients for prognostication before and/or after TAVI			
Rubin et al^21^	Patients undergoing TAVI without pre-existing PPM	High-grade AVB	Immediately before TAVI Immediately after TAVI
Akin et al^22^	Patients undergoing TAVI without pre-existing PPM	High-grade AVB Abnormal EPS	Immediately before TAVI Immediately after TAVI 7 d after TAVI
Eksik et al^23^	Patients undergoing TAVI without pre-existing PPM	High-grade AVB	Immediately before TAVI Immediately after TAVI
Rivard et al^24^	Patients undergoing TAVI without pre-existing PPM	High-grade AVB	Immediately before TAVI Immediately after TAVI
Shin et al^25^	Patients undergoing TAVI without pre-existing PPM	High-grade AVB	Immediately before TAVI Immediately after TAVI
Eksik et al^26^	Patients undergoing TAVI without pre-existing PPM	High-grade AVB	Immediately before TAVI Immediately after TAVI
Kostopoulou et al^27^	Patients undergoing TAVI without pre-existing PPM	High-grade AVB New LBBB plus abnormal EPS	Immediately before TAVI 2 d after TAVI
Lopez-Aguilera et al^28^	Patients undergoing TAVI without pre-existing PPM	High-grade AVB	Immediately before TAVI 30 min after TAVI
Badenco et al^30^	Patients undergoing TAVI without pre-existing PPM	High-degree AVB Abnormal EPS	Immediately before TAVI Immediately after TAVI 2 d after TAVI for Edwards Sapien and 5 d after procedure for CoreValve
Makki et al^31^	Patients undergoing TAVI without pre-existing PPM but underwent in-hospital PPM implantation	LBBB and abnormal EPS	Performed a median of 6 (range 2–210) d after TAVI
Krishnaswamy et al^34^	Patients undergoing TAVI in the absence of pre-existing PPM, AF, or persistent intraprocedural AVB	High-grade AVB	Immediately after TAVI
Reiter et al^35^	Patients undergoing TAVI without pre-existing pacemaker or persistent AF	High-grade AVB	Immediately before TAVI After balloon predilation Immediately after TAVI
Ferreira et al^38^	Patients undergoing TAVI without pre-existing PPM	High-grade AVB Abnormal EPS	Day 1–7 before TAVI Day 4–5 after TAVI
EPS performed in patients with equivocal pacing indication post-TAVR			
Tovia-Brodie et al^29^	Patients undergoing TAVI without pre-existing PPM plus one of the following: New-onset LBBB Old LBBB and PR increase >20 ms Slow AF(<100/min) in presence of old or new-onset LBBB	Abnormal EPS	After TAVI (median 6 d)
Rogers et al^32^	Patients with equivocal indication for pacing after TAVI (high-degree AVB, LBBB, sinus nodal dysfunction, other)	Abnormal EPS	Before hospital discharge (>24 h post-TAVI)
Knecht et al^33^	Patients with LBBB (new or pre-existing) undergoing TAVI	Abnormal EPS	<24 h after TAVI
Bourenane et al^36^	Patients with equivocal indication for pacing after TAVI (LBBB, transient AVB, other)	Abnormal EPS	2–5 d after TAVI
Nauchi et al^37^	Patients with transient AVB after TAVI	Abnormal EPS	During hospitalization after TAVI

AF = atrial fibrillation; AVB = atrioventricular block; LBBB = left bundle branch block; PPM = permanent pacemaker; other abbreviations as in Table 1.

In 10 of the included studies, EPS parameters were used to guide PPM decisions post-TAVI.^29^^,^^32^^,^^33^^,^^36^^,^^37^ In 5 of these studies, EPS was only performed among patients with equivocal post-TAVR pacing indication. The indications for EPS were new or pre-existing LBBB in 4 studies and transient intraprocedural HG-AVB in 3 studies. Other miscellaneous reasons, including sinus nodal dysfunction, were also considered in 2 of these studies. The criteria for PPM were prolonged HV interval (threshold varying from 55 to 75 ms per study) in 3 studies and induction of intrahisian or infrahisian block in 3 studies. In the remaining 5 studies in which EPS was performed in all patients undergoing TAVI,^22^^,^^27^^,^^30^^,^^31^^,^^38^, the criteria for PPM implantation were new LBBB plus prolonged HV interval in 3 studies and isolated prolonged HV interval in 2 studies (threshold varying from 55 to 80 m across studies).

### Changes of EPS parameters before and after TAVI

**Figure 1:**
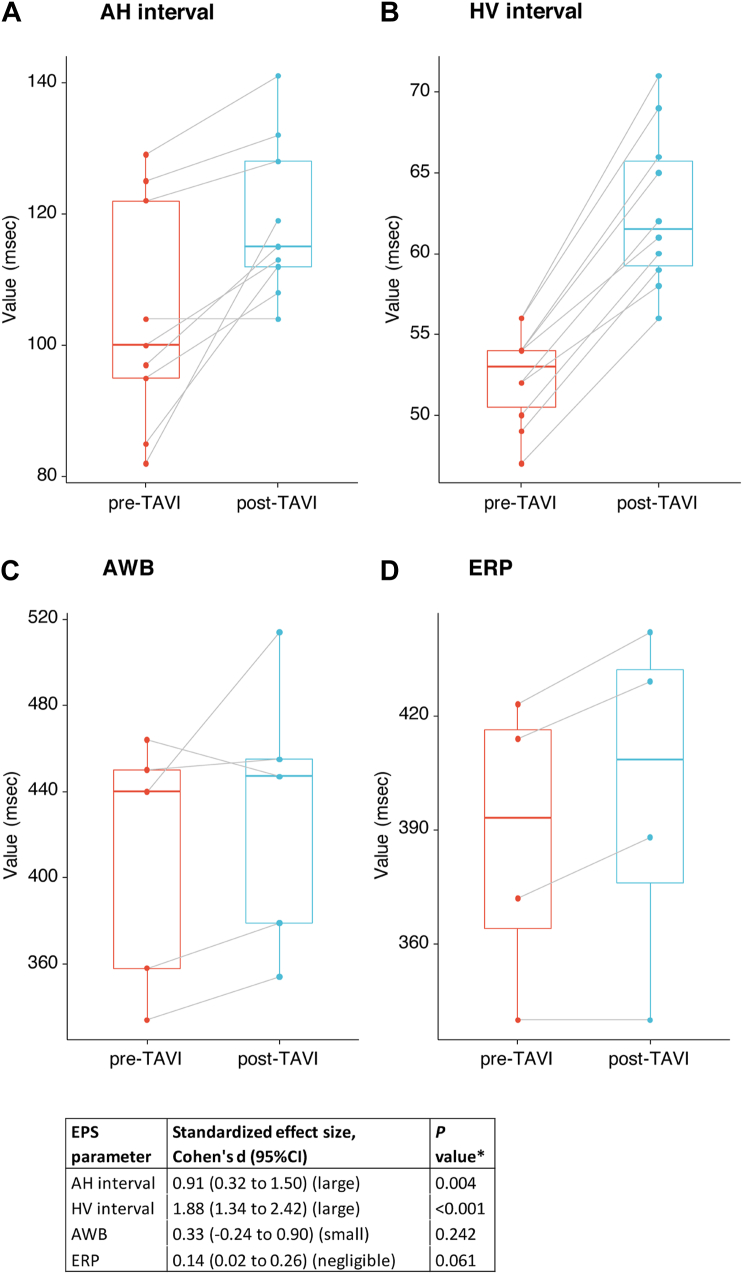
Summary mean changes of atrioventricular conduction parameters before and after transcatheter aortic valve implantation (TAVI). **A:** Atrium to His (AH) interval. **B:** His to ventricle (HV) interval. **C:** Anterograde Wenckebach cycle length (AWB). **D:** Effective refractory period (ERP) (atrioventricular node). ∗Paired sample *t* test. CI = confidence interval; EPS = electrophysiological study.

Data on EPS parameters before and after TAVI (in patients without immediate AVB) were available for the AH interval, HV interval, anterograde Wenckebach cycle length, and anterograde AV nodal effective refractory period. The changes in these parameters in each study are summarized in Figure 1. Changes were consistent across studies, with the majority showing an increase in the absolute values of all intervals after TAVI. The changes were statistically significant and of large magnitude for the AH interval (mean pre-TAVI 104 ms; mean post-TAVI 119 ms; Cohen’s d 0.91; *P* = .004) and the HV interval (mean pre-TAVI 52 ms; mean post-TAVI 63 ms; Cohen’s d 1.88; *P* <.001).

### EPS parameters as predictors of HG-AVB post-TAVI

Across 12 studies in which a PPM was implanted for a standard, unequivocal indication (HG-AVB), a total of 153 of 957 patients (16%) received a PPM. The timing of HG-AVB and PPM implantation was before hospital discharge in most studies, although occasionally it occurred up to 30 days post-TAVI. PPM implantation rates for HG-AVB ranged from 4% to 32% among studies (Table 3).

**Table 3:** PPM implantation for high-grade AVB after TAVI

Study	No. of patients	PPM implanted for high-grade AVB	Timing of AVB and PPM implantation
Rubin et al^21^	18	4 (22)	3 before hospital discharge and 1 at 10 d
Akin et al^22^	45	10 (22)	Within 7 d post-TAVI
Eksik et al^23^	28	1 (4)	Before hospital discharge
Rivard et al^24^	75	14 (19)	Median 2 d (range 0–30)
Shin et al^25^	25	8 (32)	Before hospital discharge
Eksik et al^26^	55	8 (15)	Before hospital discharge
Kostopoulou et al^27^	30	7 (23)	Median 2 d post-TAVI (range 2–24)
Lopez-Aguilera et al^28^	131	33 (25)	Within 72 h post-TAVI
Badenco et al^30^	84	17 (20)	Before hospital discharge
Krishnaswamy et al^34^	284	19 (7)	N/A
Reiter et al^35^	108	16 (15)	Within 30 d post-TAVI
Ferreira et al^38^	74	16 (22)	Within 5 d post-TAVI

Data are given absolute number (n) with percentage (%) as reported in the primary studies.

Abbreviations as in Tables 1 and 2.

**Figure 2:**
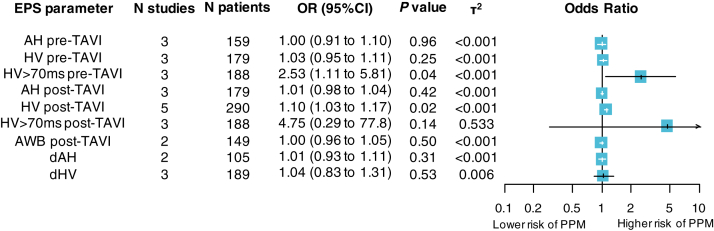
Random-effects meta-analysis of EPS-derived predictors for permanent pacemaker implantation following TAVI. All estimates are reported per millisecond of change of the EPS parameter except for HV >70 ms pre-TAVI and HV >70 ms post-TAVI, which are shown as categorical estimates. dAH = delta AH; dHV = delta HV; OR = odds ratio; PPM = permanent pacemaker; TAVR = transcatheter aortic valve replacement; other abbreviations as in Figure 1.

Figure 2 and Supplemental Material Section 3 show the random-effects summary estimates for each examined EPS parameter in association with post-TAVI HG-AVB requiring PPM. The pre-TAVI HV interval >70 ms was significantly associated with an increased risk of HG-AVB and PPM implantation (OR 2.53; 95% CI 1.11–5.81; *P* = .04; heterogeneity τ^2^ <0.001). Furthermore, among patients without immediate AVB, the absolute value of the HV interval post-TAVI was also statistically significantly associated with subsequent HG-AVB and PPM requirement (OR 1.10; 95% CI 1.03–1.17; *P* = .02; τ^2^ <0.001; per 1-ms increase). Other parameters, including pre- and post-TAVI AH, anterograde Wenckebach cycle length, delta AH, and delta HV, did not show significant associations with post-TAVI HG-AVB.

### EPS parameters to guide PPM implantation post-TAVI

In 10 studies (506 patients), PPM implantation decision-making was guided by post-TAVI EPS findings. Table 4 lists the EPS criteria composing the indications for PPM implantation in each study. In these studies, the prevalence of post-TAVI LBBB ranged from 21% to 100%, and the prevalence of intraprocedural transient AVB ranged from 9% to 100% (Table 4). A total of 124 of 506 patients (25%) received a PPM for that indication before hospital discharge.

**Table 4:** PPM implantation guided by abnormal EPS

Study	No. of patients	No. with LBBB post-TAVR	No. with transient intraoperative AVB	Criteria for PPM implantation	Timing of PPM implantation	PPM implanted for abnormal EPS	Long-term PPM dependency in patients implanted for abnormal EPS	Definition of PPM dependency
Akin et al^22^	45	20 (44)	N/A	New LBBB plus HV ≥75 ms	Within 7 d post-TAVI	13 (29)	Not assessed	N/A
Kostopoulou et al^27^	30	14 (47)	N/A	New LBBB plus HV >70 ms	Median 2 d post-TAVI (range 2-24)	1 (3)	1/1 (100)	Asystole or HG-AVB with or without escape rhythm after cessation of pacing
Tovia-Brodie et al^29^	26	81 (100)	N/A	Intrahisian block HV interval ≥75 ms Second-degree infranodal block during incremental atrial pacing at a cycle length <400 ms	N/A	8 (31)	Not assessed	N/A
Badenco et al^30^	84	30 (36)	13 (15)	HV interval >80 m	Before hospital discharge	9 (11)	1/9 (11)	Persistent HG-AVB
Makki et al^31^	7	5 (71)	N/A	LBBB and HV interval >55 ms or elicitation of complete heart block	N/A	7 (100)	1/7 (14)	(1) >50% pacing on PPM interrogation (2) Underlying HG-AVB (3) Underlying asystole >5 s (4) Symptoms in the setting of bradycardia (rate <50 bpm)
Rogers et al^32^	95	20 (21)	N/A	Intrahisian or infrahisian block with decremental atrial pacing with or without isoproterenol challenge	N/A	28 (29)	Not assessed	N/A
Knecht et al^33^	56	56 (100)	N/A	HV interval >55 ms	N/A	15 (27)	8/15 (53)	HG-AVB on 12-lead ECG and/or ventricular pacing >1% despite algorithms to minimize pacing
Bourenane et al^36^	78	63 (81)	7 (9)	HV interval >70 ms High-grade infrahisian block during incremental atrial pacing at rate ≤100 bpm	N/A	35 (45)	27/35 (77)	Ventricular pacing >1%
Nauchi et al^37^	11	N/A	11 (100)	Induction of AVB with RV apical pacing at 100 per min for 1 min with or without IV procainamide (10 mg/kg) for 10 min	N/A	3 (27)	2/3 (67)	Persistent HG-AVB
Ferreira et al^38^	74	33 (45)	N/A	HV interval ≥95 ms High-grade infrahisian block during atrial pacing at rate ≤150 bpm	Within 5 d post-TAVI	5 (7)	Not assessed	N/A

Values are given as absolute number (n) with percentage (%) as reported in the primary studies.

ECG = electrocardiogram; HG-AVB = high-grade atrioventricular block; IV = intravenous; RV = right ventricle; other abbreviations as in Tables 1 and 2.

Six studies reported rates of pacemaker dependency during follow-up among patients receiving PPM for abnormal EPS findings. The definitions of pacemaker dependency in each of the 6 studies are listed in Table 4. Of 70 patients, 40 (57%) were PPM-dependent during variable follow-up ranging from 7 days to 17 months in different studies. Furthermore, 6 studies reported that the postdischarge rate of sudden cardiac death or PPM implantation for HG-AVB among patients with a negative post-TAVI EPS was 1.7% (4/229 patients) during follow-up of 3–12 months.

## Discussion

PPM implantation for HG-AVB after TAVI is associated with longer hospitalization, higher readmission rates, and possibly increased morbidity and mortality.^7^^,^^39^^,^^40^ Electrocardiographic predictors of post-TAVI AVB based on the preprocedure ECG, as well as procedural and anatomic characteristics, can guide procedural planning and patient counseling for PPM risk.^7^^,^^8^ However, uncertainty exists regarding the management of patients with equivocal PPM indications after TAVI. This uncertainty is reflected by the broad range of guidance in the 2020 ACC Expert Consensus Decision Pathway on Management of Conduction Disturbances in Patients Undergoing Transcatheter Aortic Valve Replacement wherein “monitoring, and consideration for EPS and PPM are advised for patients with new, progressive or pre-existing conduction disturbance that changes post-procedure.”^41^

In this meta-analysis of 18 studies reporting the value of peri-TAVI EPS to predict HG-AVB, we found the rate of PPM implantation for HG-AVB was 16%. The AH and HV intervals showed the most consistent absolute increases after TAVI. The HV interval pre- and post-TAVI was significantly associated with subsequent HG-AVB and PPM requirement. Furthermore, among patients without early HG-AVB who received a PPM for abnormal EPS findings after TAVI, half were PPM-dependent during posthospitalization follow-up. Among patients with a normal EPS after TAVI who did not receive a PPM, the rate of sudden cardiac death or HG-AVB after hospital discharge was very low.

Most PPM implantations after TAVI are unavoidable and clearly indicated. However, some patients with new AV conduction disease without definite PPM indications may receive a prophylactic PPM because of concern for progression to higher-grade AVB, and many more patients undergo prolonged ambulatory rhythm monitoring after hospital discharge. Up to 10% of patients without an immediate PPM indication may develop delayed, posthospitalization HG-AVB, with first-degree AVB and bundle branch blocks being predictors of delayed AVB.^9^^,^^42^ However, in a study using 30-day continuous ambulatory monitoring in post-TAVI patients, only 14% of patients with new LBBB progressed to second- or third-degree AVB.^43^ Therefore, EP testing may offer useful information in refining risk stratification for patients before TAVI but also for those with equivocal PPM indications post-TAVI, such as new LBBB or right bundle branch block with or without first-degree AVB or atrial fibrillation, and transient intraprocedural HG-AVB.

The current analysis allows synthesis of evidence across studies with diverse patient populations and practice patterns and amplifies the statistical power to detect associations between EPS parameters and TAVI-related AVB. We found the pre- and post-TAVI HV interval was a significant predictor of AVB and PPM requirement. The HV interval is an integral measure of intrahisian and infrahisian system function, with an interval ≥55 ms considered abnormal. We found less robust evidence for the pre- or post-TAVI AH interval. This is not surprising considering that the compact AV node and its fast pathway input are less likely to be injured during TAVI, as opposed to the His bundle and proximal left bundle branch.^44^ Furthermore, unlike the HV interval, the AH interval varies significantly depending on autonomic input, thus providing a less reproducible measure of AV conduction status.

The approach of EPS-guided PPM implantation after TAVI was specifically tested in 10 of the included studies. Using various criteria to define abnormal EPS, this approach resulted in 1 in 4 patients receiving a PPM after EPS before hospital discharge. Outside of the TAVI setting, HV >70 ms in patients with syncope and bundle branch block is an indication for a PPM.^45^ Similarly, pacing- or procainamide-induced infrahisian block is a PPM indication in most settings. Two of the studies in this meta-analysis used an HV interval threshold of 55 ms to recommend PPM implantation. It is reasonable to individually consider PPM for HV interval between 55 and 70 ms depending also on other patient-specific factors and preferences. Even though the pre-TAVI HV interval had prognostic significance in our study, pre-TAVI EPS is not included in the proposed algorithm because it would be unlikely to impact management unless AV conduction changes occur after valve deployment.

More than half of the patients who received a PPM for an abnormal EPS were pacemaker-dependent (defined as persistent HG-AVB or non-negligible ventricular pacing percentage among studies) when long-term follow-up PPM data were available. This is a large value that supports the use of EPS-guided PPM implantation in some patients. PPM utilization could be improved by fine-tuning patient criteria and EPS parameter thresholds to increase their specificity for subsequent HG-AVB. In addition, conduction changes after TAVI can evolve over a period of days, particularly for self-expanding valves, and the optimal timing of EPS after TAVI needs further investigation. Noteworthy, the very low rate of subsequent PPM requirement or sudden cardiac death in patients with reassuring EPS results who did not receive a PPM before hospital discharge further highlights the potential value of peri-TAVR EPS.

The implications of EPS on TAVI-related costs and resource utilization merit consideration. Payment and reimbursement models for TAVI vary across health systems, and the cost-effectiveness of EPS requires further study. There is also a theoretical concern for prolonged hospital stay in patients undergoing EPS after TAVI. However, in one of the largest studies by Rogers et al^32^ included in this meta-analysis, patients with a negative EPS had comparable length of hospital stay as patients without any conduction disturbance. Similarly, Krishnaswamy et al^34^ reported a similar length of hospital stay in patients with positive and negative EPS. Selective use of EPS when it can meaningfully impact decision-making ultimately may reduce costs of care and adverse outcomes by reducing over- and underutilization of ambulatory rhythm monitoring and PPM. However, because of the heterogeneity in existing evidence, the current data do not support the broad adoption of EPS-guided decision-making in clinical practice. Further research is needed to determine actionable thresholds of key AV conduction parameters, optimal EPS protocols, and patient subgroups who will benefit the most.

### Study limitations

The included studies had variations in procedural characteristics, EPS protocols and timing, and actionable thresholds of EPS parameters. Similarly, there was a mix of patients undergoing self-expanding and balloon-expandable TAVI in the cumulative data analyzed. The value of EPS in predicting short- and longer-term PPM requirement likely differs in the 2 groups. We did not have the data required to investigate the value of EPS parameters in different subgroups, including those with various pre- or post-TAVI conduction abnormalities (such as LBBB). The overlap in enrollment periods across studies and the limited or absent study-level information on EPS parameters stratified by the different TAVI systems and implantation techniques did not allow us to investigate the value of EPS parameters over time in correlation with evolving TAVI technology and techniques. Furthermore, with the exception of the small randomized study by Kostopoulou et al,^27^ all other included studies were observational and findings may have been affected by confounders. Large randomized trials of an EPS-guided vs conventional approach to post-TAVI PPM implantation are needed to inform on the outcomes of patients with equivocal pacing indications after TAVI.

## Conclusion

Invasive EPS parameters can offer useful insights in the risk stratification for HG-AVB after TAVI. Selective utilization of EPS for assessment of the AV conduction system in patients with borderline PPM indications after TAVI can be considered within the context of the limitations of the currently available data. Future randomized studies comparing EPS-guided and standard-of-care approaches in patients with new, non–high-grade AV conduction disturbances after TAVI are needed to definitively assess the impact on patient outcomes, resource utilization, and costs before broader adoption of EPS in the peri-TAVI setting can be justified.
