# Supplementary material for: Invasive electrophysiological testing to predict and guide permanent pacemaker implantation after transcatheter aortic valve implantation: A meta-analysis
Source: Heart Rhythm O2. 2022 Oct 22;4(1):24–33. doi: 10.1016/j.hroo.2022.10.007 (PMC9877393; doi:10.1016/j.hroo.2022.10.007)

SUPPLEMENTARY MATERIAL

Supplementary section 1: Search strategy.

	Query	Results*
**MEDLINE**		
**#1**	Search (transcatheter OR transaortic OR transfemoral OR transarterial OR transapical OR transluminal OR percutaneous OR "catheter based")	189690
**#2**	Search (aorta OR aortic)	316106
**#3**	Search (replace OR replacement* OR implant OR implantation* OR insertion*)	1085312
**#4**	Search (#1 AND #2 AND #3)	14971
**#5**	Search (TAVI OR TAVR)	7310
**#6**	Search ("transcatheter aortic valve replacement" OR "transcatheter aortic valve implantation" OR "percutaneous aortic valve" OR "catheter based aortic valve" OR (transcath* AND aort* AND valv* AND (replace* OR implant*)) OR (catheter AND aort* AND valve*))	13718
**#7**	Search "transcatheter aortic valve replacement"[MeSH Terms]	5209
**#8**	Search (#4 OR #5 OR #6 OR #7)	17567
**#9**	Search ("electrophysiological study" OR "electrophysiological studies" OR (electrophysiologic* AND stud*) OR eps)	78439
**#10**	Search (((cardiac electrophysiologic study[MeSH Terms]) OR electrophysiologic study, cardiac[MeSH Terms]) OR electrophysiologic studies, cardiac[MeSH Terms]) OR cardiac electrophysiologic studies[MeSH Terms] OR ((electrogram, intracardiac[MeSH Terms]) OR electrograms, intracardiac[MeSH Terms]) OR intracardiac electrophysiologic technique[MeSH Terms]	6246
**#11**	Search (intracardiac AND (electrogra* OR electrocardiogra* OR electrophysiolog*))	3106
**#12**	Search (#9 OR #10 OR #11)	83323
**#13**	Search (#8 AND #12)	141
**EMBASE**		
**#1**	‘TAVI'/exp OR 'TAVR'/exp OR 'percutaneous aortic valve implantation'/exp OR 'percutaneous aortic valve implantation' OR 'percutaneous aortic valve replacement'/exp OR 'percutaneous aortic valve replacement' OR 'trans-apical aortic valve implantation'/exp OR 'trans-apical aortic valve implantation' OR 'trans-apical aortic valve replacement'/exp OR 'trans-apical aortic valve replacement' OR 'trans-arterial aortic valve implantation'/exp OR 'trans-arterial aortic valve implantation' OR 'trans-arterial aortic valve replacement'/exp OR 'trans-arterial aortic valve replacement' OR 'trans-catheter aortic valve implantation'/exp OR 'trans-catheter aortic valve implantation' OR 'trans-catheter aortic valve replacement'/exp OR 'trans-catheter aortic valve replacement' OR 'trans-cutaneous aortic valve implantation'/exp OR 'trans-cutaneous aortic valve implantation' OR 'trans-cutaneous aortic valve replacement'/exp OR 'trans-cutaneous aortic valve replacement' OR 'trans-femoral aortic valve implantation'/exp OR 'trans-femoral aortic valve implantation' OR 'trans-femoral aortic valve replacement'/exp OR 'trans-femoral aortic valve replacement' OR 'transapical aortic valve implantation'/exp OR 'transapical aortic valve implantation' OR 'transapical aortic valve replacement'/exp OR 'transapical aortic valve replacement' OR 'transarterial aortic valve implantation'/exp OR 'transarterial aortic valve implantation' OR 'transarterial aortic valve replacement'/exp OR 'transarterial aortic valve replacement' OR 'transcatheter aortic valve implantation'/exp OR 'transcatheter aortic valve implantation' OR 'transcatheter aortic valve replacement'/exp OR 'transcatheter aortic valve replacement' OR 'transcutaneous aortic valve implantation'/exp OR 'transcutaneous aortic valve implantation' OR 'transcutaneous aortic valve	23294

	replacement'/exp OR 'transcutaneous aortic valve replacement' OR 'transfemoral aortic valve implantation'/exp OR 'transfemoral aortic valve implantation' OR 'transfemoral aortic valve replacement'/exp OR 'transfemoral aortic valve replacement'	
**#2**	‘cardiac electrophysiology'/exp OR 'cardiac electrophysiology' OR 'electrophysiology, heart'/exp OR 'electrophysiology, heart' OR 'heart electrophysiology'/exp OR 'heart electrophysiology' OR 'electrophysiological study' OR 'electrophysiological studies' OR 'electrophysiologic study' OR 'electrophysiologic studies' OR 'electrophysiolog* stud*' OR 'intracardiac electrocardiography'/exp OR 'intracardiac electrocardiography' OR 'intracardiac electrogram'/exp OR 'intracardiac electrogram'	275156
**#3**	#1 AND #2	1458
**#4**	#3 AND ([article]/lim OR [article in press]/lim) AND [humans]/lim AND [clinical study]/lim	484
**CENTRAL**		
**#1**	(transcatheter):ti,ab,kw OR (transfemoral):ti,ab,kw OR (transaortic):ti,ab,kw OR (transapical):ti,ab,kw OR (transluminal):ti,ab,kw	5153
**#2**	(transarterial):ti,ab,kw OR (percutaneous):ti,ab,kw OR (catheter?based):ti,ab,kw	19622
**#3**	#1 OR #2	21963
**#4**	(aortic):ti,ab,kw AND (valve OR prosthesis):ti,ab,kw	2933
**#5**	(replace* OR implant* OR insert*):ti,ab,kw	83943
**#6**	#3 AND #4 AND #5	844
**#7**	MeSH descriptor: [Transcatheter Aortic Valve Replacement] explode all trees	136
**#8**	TAVR OR TAVI OR "transcatheter aortic valve replacement" OR "transcatheter aortic valve implantation" OR ((trans* OR catheter OR percutaneous) AND (aort* AND valv*))	1673
**#9**	#6 OR #7 OR #8	1686
**#10**	("electrophysiologic studies"):ti,ab,kw OR ("electrophysiological studies"):ti,ab,kw OR ("electrophysiologic study"):ti,ab,kw OR ("electrophysiological study"):ti,ab,kw OR (eps):ti,ab,kw	1259
**#11**	electrophysiolog* stud*	5142
**#12**	("intracardiac electrogram"):ti,ab,kw OR ("intracardiac electrocardiography"):ti,ab,kw	23
**#13**	MeSH descriptor: [Electrophysiologic Techniques, Cardiac] explode all trees	190
**#14**	#10 OR #11 OR #12 OR #13	6000
**#15**	#9 AND #14	13

Supplementary section 2: Study selection flowchart.

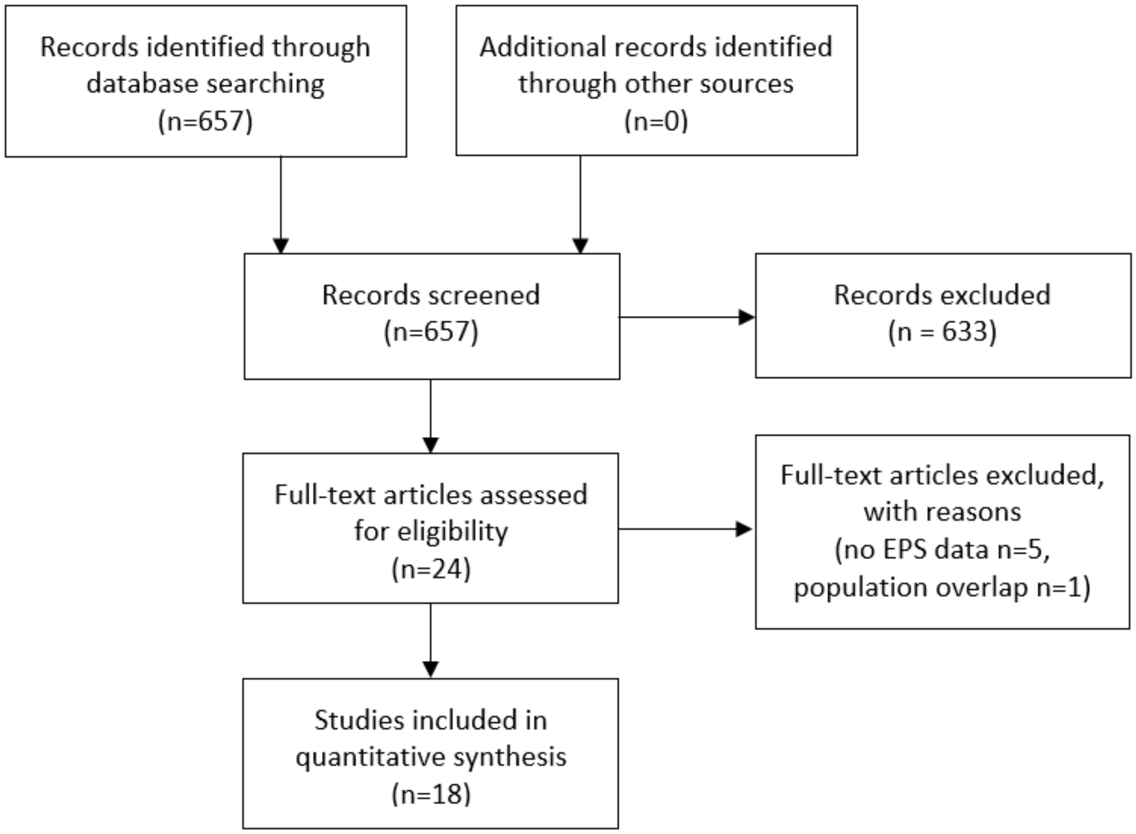

Supplementary section 3: EPS parameter-specific random-effect meta-analysis as predictors of PPM implantation.

TE corresponds to the natural logarithm of point estimate and seTE to the natural logarithm of standard error of the effect.

AH pre TAVR

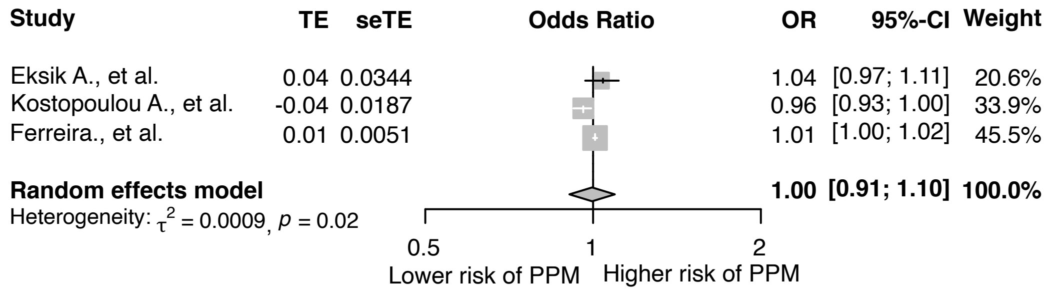

HV pre TAVR

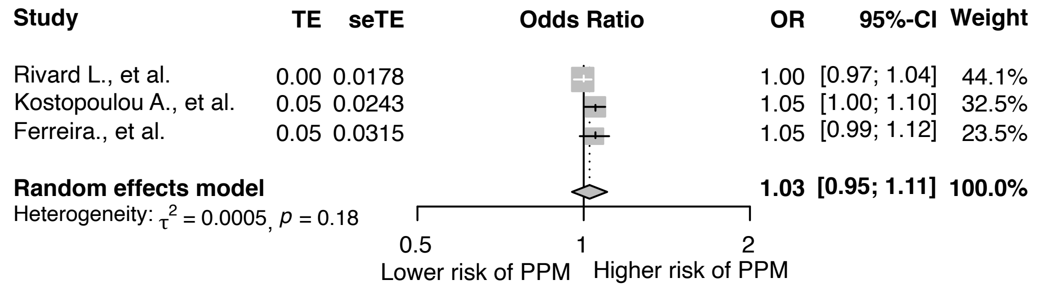

HV >70msec pre TAVR

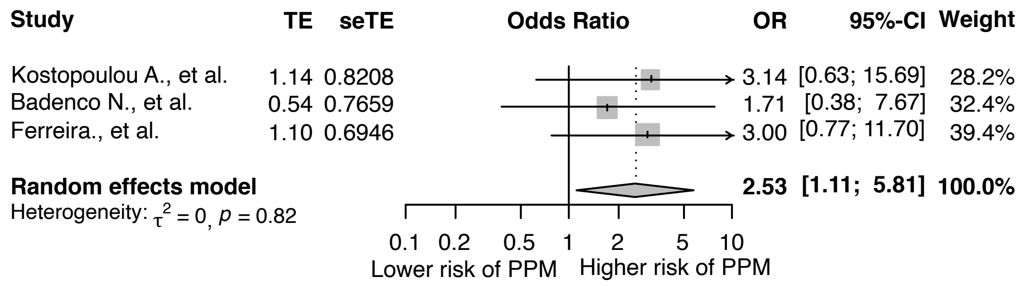

AH post TAVR

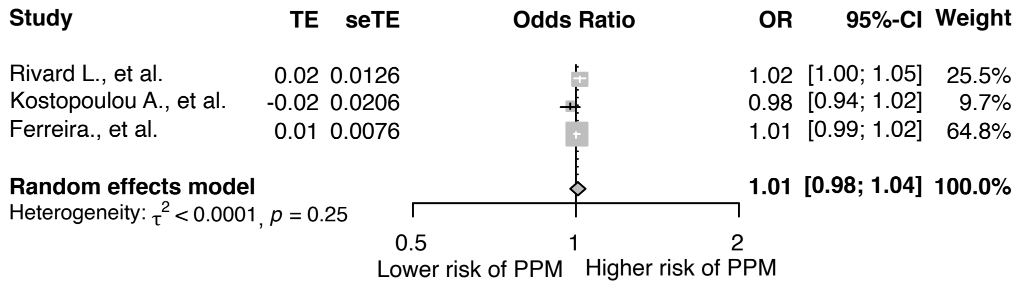

HV post TAVR

Study	TE	seTE	OR	95%-CI	Weight
Rivard L., et al.	0.03	0.0124	1.03	[1.01; 1.06]	34.7%
Eksik A., et al.	0.15	0.0683	1.16	[1.01; 1.33]	11.7%
Kostopoulou A., et al.	0.12	0.0630	1.13	[1.00; 1.28]	13.0%
Knecht S., et al.	0.10	0.0365	1.11	[1.03; 1.19]	22.9%
Ferreira., et al.	0.14	0.0490	1.15	[1.04; 1.27]	17.6%
**Random effects model**			**1.10**	**[1.03; 1.17]**	**100.0%**

Heterogeneity: $\tau^2 = 0.0021$, $p = 0.02$

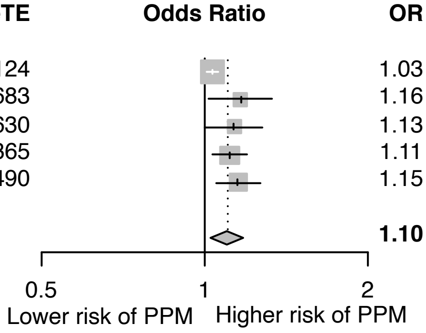

HV >70msec post TAVR

Study	TE	seTE	OR	95%-CI	Weight
Kostopoulou A., et al.	2.13	1.2352	8.43	[0.75; 94.89]	24.3%
Badenco N., et al.	0.64	0.6995	1.90	[0.48; 7.49]	48.9%
Ferreira., et al.	2.71	1.1547	15.00	[1.56; 144.21]	26.8%
**Random effects model**			**4.75**	**[0.29; 77.79]**	**100.0%**

Heterogeneity: $\tau^2 = 0.5332$, $p = 0.25$

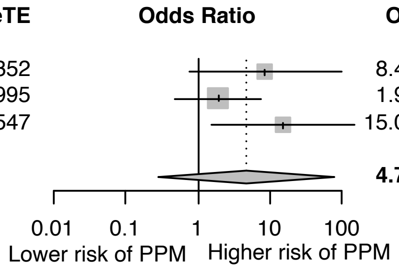

aWBP

Study	TE	seTE	OR	95%-CI	Weight
Rivard L., et al.	0.00	0.0051	1.00	[0.99; 1.01]	50.0%
Ferreira., et al.	0.01	0.0051	1.01	[1.00; 1.02]	50.0%
**Random effects model**			**1.00**	**[0.96; 1.05]**	**100.0%**

Heterogeneity: $\tau^2 = 0$, $p = 0.33$

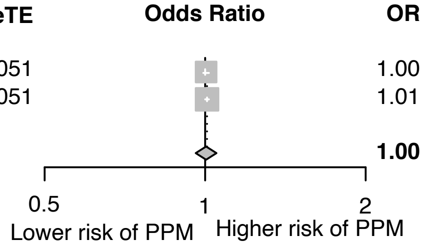

Delta AH

Study	TE	seTE	OR	95%-CI	Weight
Rivard L., et al.	0.01	0.0101	1.01	[0.99; 1.03]	85.7%
Kostopoulou A., et al.	0.03	0.0248	1.03	[0.98; 1.08]	14.3%
**Random effects model**			**1.01**	**[0.93; 1.10]**	**100.0%**

Heterogeneity: $\tau^2 = 0$, $p = 0.46$

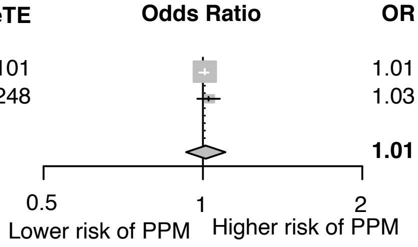

Delta HV

Study	TE	seTE	OR	95%-CI	Weight
Rivard L., et al.	0.14	0.0400	1.15	[1.06; 1.24]	35.0%
Kostopoulou A., et al.	-0.05	0.0749	0.95	[0.82; 1.10]	23.3%
Badenco N., et al.	0.01	0.0178	1.01	[0.98; 1.05]	41.7%
**Random effects model**			**1.04**	**[0.83; 1.31]**	**100.0%**

Heterogeneity: $\tau^2 = 0.0064$, $p < 0.01$

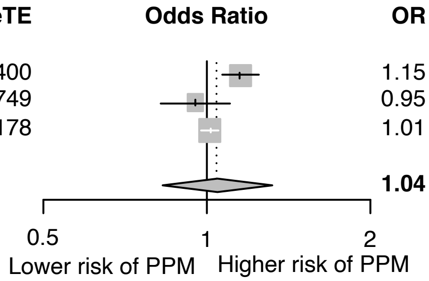

Supplement: Supplementary Material [file mmc1.pdf]
